# Sumoylation of eIF4A2 affects stress granule formation

**DOI:** 10.1242/jcs.184614

**Published:** 2016-06-15

**Authors:** Jirapas Jongjitwimol, Robert A. Baldock, Simon J. Morley, Felicity Z. Watts

**Affiliations:** 1 Genome Damage and Stability Centre, School of Life Sciences, University of Sussex, Falmer, Brighton BN1 9RQ, UK; 2 Department of Biochemistry and Biomedical Science, School of Life Sciences, University of Sussex, Falmer, Brighton BN1 9QG, UK

**Keywords:** SUMO, Arsenite, Hippuristanol, Heat shock, Ionising radiation, Translation, Protein synthesis

## Abstract

Regulation of protein synthesis is crucial for cells to maintain viability and to prevent unscheduled proliferation that could lead to tumorigenesis. Exposure to stress results in stalling of translation, with many translation initiation factors, ribosomal subunits and mRNAs being sequestered into stress granules or P bodies. This allows the re-programming of the translation machinery. Many aspects of translation are regulated by post-translational modification. Several proteomic screens have identified translation initiation factors as targets for sumoylation, although in many cases the role of this modification has not been determined. We show here that eIF4A2 is modified by SUMO, with sumoylation occurring on a single residue (K226). We demonstrate that sumoylation of eIF4A2 is modestly increased in response to arsenite and ionising radiation, but decreases in response to heat shock or hippuristanol. In arsenite-treated cells, but not in hippuristanol-treated cells, eIF4A2 is recruited to stress granules, suggesting sumoylation of eIF4A2 correlates with its recruitment to stress granules. Furthermore, we demonstrate that the inability to sumoylate eIF4A2 results in impaired stress granule formation, indicating a new role for sumoylation in the stress response.

## Abstract (precis)

**Summary:** In response to stress, proteins required to initiate protein synthesis are modified; we demonstrate that sumoylation of eIF4A2 correlates with its recruitment to stress granules.

## INTRODUCTION

Protein synthesis is a fundamental cellular process, which needs to be efficiently regulated, particularly in response to environmental stresses. It comprises three stages: initiation, elongation and termination. Of these, the initiation step has a major regulatory role in protein synthesis, and affects not only the level of protein synthesis, but also which mRNAs are translated (Sonenberg and Hinnebusch, 2009; Guertin and Sabatini, 2007; Laplante and Sabatini, 2012; Morley et al., 2005; Jackson et al., 2010). Translation initiation involves the binding of the preinitiation complex [comprising the 40S ribosomal subunit, eukaryotic initiation factor 3 (eIF3), eIF1A, eIF1, eIF5 and eIF2 (eIF2-GTP-methionyl initiator tRNA)] to capped mRNA that is bound by eIF4F (a complex of eIF4A, eIF4E and eIF4G). The resulting 48S preinitiation complex then scans the 5′ untranslated region of the mRNA for the initiation codon, at which point early initiation factors are released and the large ribosomal subunit is recruited, allowing protein synthesis to begin (reviewed in Jackson et al., 2010).

In response to stress, most of the protein synthesis within a cell is shut down in order to conserve energy to allow repair of stress-induced damage and reprogramming of the translational machinery (e.g. Spriggs et al., 2010; Balagopal and Parker, 2009). Polysomes are disassembled, leading to the stalling of initiation complexes that are then recruited to specialised bodies, termed stress granules. These granules are proposed to be sites where, during stress and recovery, individual mRNAs are sorted for storage, degradation or translation. The granules are highly dynamic and can either fuse with P bodies that contain the mRNA decay machinery, or can release components to allow resumption of translation (Anderson and Kedersha, 2009). Formation of stress granules is induced by phosphorylation of eIF2α (Kedersha et al., 1999), or by other factors, such as conditions that inhibit eIF4A (e.g. Dang et al., 2006). Also important for induction are the T-cell internal antigen (TIA) proteins, TIA-1 and TIA-R (also known as TIAL1), that have prion-related C-terminal domains (Waris et al., 2014). In mammalian cells, stress granules comprise numerous proteins including eIF2, eIF3, eIF4A, eIF4E, eIF4G, poly(A)-binding protein (PABP), the small ribosomal subunit and mRNAs (e.g. Balagopal and Parker, 2009; Anderson et al., 2015). Depletion of any one of a number of these proteins can lead to the formation of stress granules (Mazroui et al., 2006; Mokas et al., 2009).

eIF4G is a large scaffold protein that possesses domains that interact with eIF4E and eIF4A, to form the eIF4F complex, as well as those that interact with eIF3 and PABP (Sonenberg and Hinnebusch, 2009; Laplante and Sabatini, 2012; Morley et al., 2005; Jackson et al., 2010). eIF4E is an mRNA-cap-binding protein, and eIF4A proteins comprises a family of DEAD-box RNA helicases, which are involved in many aspects of RNA metabolism. In mammalian cells, there are three highly related eIF4A proteins, eIF4A1, eIF4A2 and eIF4A3, which have diverse and non-overlapping roles in mRNA metabolism (Lu et al., 2014). eIF4A3 is nuclear (Chan et al., 2004), whereas eIF4A1 and eIF4A2 are cytoplasmic and, although functionally interchangeable *in vitro*, are not functionally redundant *in vivo* (Nielsen and Trachsel, 1988; Galicia-Vázquez et al., 2012; Yoder-Hill et al., 1993). Whereas both eIF4A1 and eIF4A2 interact with eIF4G, eIF4A2, but not eIF4A1, binds cNOT7 (Meijer et al., 2013). cNOT7 is a member of the CCR4–NOT complex, that is required for deadenylation of mRNA, and for microRNA (miRNA)-mediated gene regulation, indicating a specific role for eIF4A2 in this process.

The interactions and functions of the proteins in the eIF4F complex are regulated by a number of post-translational modifications. For example, during normal protein synthesis, the activity of eIF4F is regulated by eIF4E-binding proteins (4E-BPs) (Matsuo et al., 1997). 4E-BPs bind to eIF4E and compete for the interaction with eIF4G, thus inhibiting translation initiation. In mammalian cells, regulation of the interaction between 4E-BPs and eIF4E occurs through the activation of the mechanistic target of rapamycin complex 1 (mTORC1) that leads to the multi-site phosphorylation of 4E-BP1 (reviewed in Sonenberg and Hinnebusch, 2009). This prevents 4E-BP1 (also known as EIF4EBP1) from binding to eIF4E, thereby allowing formation of the eIF4F initiation complex and ribosomal recruitment of mRNA. Another post-translational modification that regulates the interaction of eIF4E with eIF4G is sumoylation of eIF4E by SUMO1 (Xu et al., 2010).

SUMO is a small ubiquitin-like modifier that can be covalently attached to target proteins (Hannoun et al., 2010). Modification by SUMO affects protein–protein interactions, protein localisation and protein activity, or can target proteins for ubiquitin-mediated proteolysis (Enserink, 2015; Geiss-Friedlander and Melchior, 2007; Watts, 2004, 2007). In many cases, it acts by providing an altered binding surface on the target protein. In mammalian cells there are three SUMO proteins, SUMO1, SUMO2 and SUMO3. SUMO2 and SUMO3 are 97% identical and are capable of forming SUMO chains, whereas SUMO1 is less similar (50% identical) and unable to form chains, but can act as a chain terminator (Matic et al., 2008). SUMO is produced as a precursor protein, which is processed to the mature form by one of a number of specific proteases. It is then activated by interaction with the E1 SUMO-activating enzyme (SAE; a complex of SAE1 and UBA2), from where it is passed to an E2, SUMO-conjugating enzyme. From here, it can be attached directly to target proteins, although in some cases conjugation requires the activity of one of a small number of SUMO ligases. Several proteomic screens have identified many of the translation initiation factors as sumoylation targets (e.g. Matafora et al., 2009; Bruderer et al., 2011; Blomster et al., 2009; reviewed in Watts et al., 2014). However, in most cases, the effect of sumoylation on individual target protein function remains to be determined.

We have previously demonstrated that fission yeast and mammalian eIF4G are sumoylated (Jongjitwimol et al., 2014), and have identified two sumoylation sites in the C-terminus of human eIF4G. Our initial studies suggest that sumoylation might have a role in regulating protein synthesis in response to stress. To further investigate this, we have analysed the effects of arsenite, heat shock, hippuristanol and ionising radiation on the sumoylation of members of the human eIF4F complex. We report here the sumoylation of eIF4A *in vivo* by both SUMO1 and SUMO2, and the identification of single sumoylation sites in eIF4A1 and eIF4A2. We demonstrate that modification of eIF4A2 by SUMO1 is increased in response to arsenite and ionising radiation but decreased in response to heat shock and hippuristanol. Furthermore, knocking down endogenous eIF4A2 and re-transfection with an unsumoylatable version of eIF4A2 has a detrimental effect on the formation of stress granules, indicating a role for sumoylation of eIF4A2 in its localisation in stress granules. Possible mechanisms whereby stress granule formation is affected by this modification are discussed.

## RESULTS

### eIF4A is sumoylated in mammalian cells

**Fig. 1.**
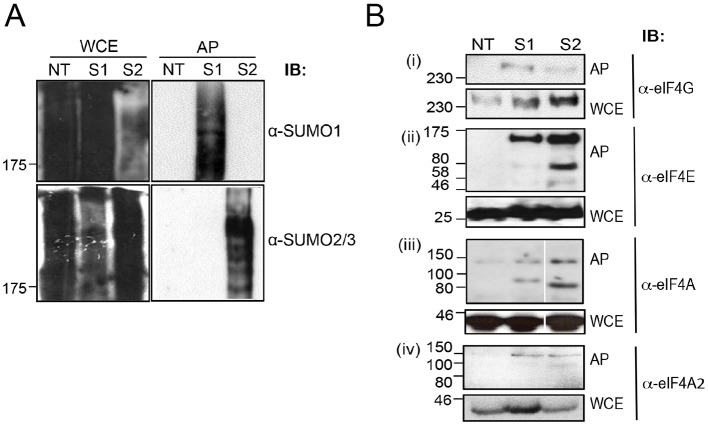
**eF4A, eIF4E and eIF4G are sumoylated in mammalian cells.** Whole-cell extracts (WCE) and affinity purification on Ni^2+^ agarose under denaturing conditions (AP) of His-tagged SUMO from non-transfected HeLa cells (NT) and HeLa cell lines stably transfected with His–SUMO1 (S1) or His–SUMO2 (S2), analysed by SDS PAGE (7.5%) and western blotted (IB) with anti-SUMO1 and anti-SUMO2/3 affinity-purified antibodies (A), or anti-eIF4G, eIF4E and eIF4A antisera and anti-eIF4A2 affinity-purified antibodies as indicated (B). The position of molecular mass markers is indicated.

The translation initiation factor eIF4F consists of three proteins: eIF4A, eIF4E and eIF4G. We and others have previously demonstrated that both mammalian eIF4E and eIF4G are sumoylated (Xu et al., 2010; Jongjitwimol et al., 2014). We therefore wished to determine whether eIF4A is also modified by SUMO. To investigate this, we affinity-purified His-tagged SUMO conjugates using Ni^2+^ agarose under denaturing conditions from cell lines stably transfected with either His–SUMO1 or His–SUMO2. Under these conditions, non-covalent interactions are disrupted allowing the identification of post-translational modifications. Western blots of affinity-purified proteins were probed with antibody against SUMO1, an antibody recognising both SUMO2 and SUMO3 (Fig. 1A) and antibody against eIF4A; eIF4E and eIF4G were used as positive controls (Fig. 1B). Western blotting with anti-eIF4G (Fig. 1Bi) identified a high molecular mass species (>250 kDa) following affinity purification of both His–SUMO1 and His–SUMO2 supporting our previous observation (Jongjitwimol et al., 2014) showing that eIF4G is modified by SUMO1 and, to a lesser extent, by SUMO2. Additionally, probing with anti-eIF4E antisera (Fig. 1Bii) identified two minor species of ∼50 and 80 kDa and a more abundant species of ∼175 kDa following purification of His–SUMO1 and His–SUMO2. Although the predicted molecular mass of SUMO is 11 kDa, it migrates on SDS-PAGE gels with an apparent molecular mass of 15–17 kDa. Thus, the minor species (∼50 and ∼80 kDa) likely represent di- and tri-sumoylated eIF4E, whereas the 175-kDa species corresponds to poly-sumoylated eIF4E. [Although SUMO1 cannot be incorporated into SUMO chains, it can act as a chain terminator (Ulrich, 2008).] These modified forms are similar in size to the previously described SUMO1-modified forms of eIF4E (Xu et al., 2010). As well as being modified by SUMO1, the results in Fig. 1Bii indicate that eIF4E is also modified by SUMO2. This would be consistent with the presence of high molecular mass poly-SUMO chains that we observe.

Having demonstrated that we are able to identify sumoylated species using denaturing conditions, we probed similar blots with anti-eIF4A antisera (Fig. 1Biii). Following affinity purification of His–SUMO, we observed species migrating with an approximate molecular mass of ∼70 and 150 kDa after purification of His–SUMO1, and His–SUMO2. Given that the anti-eIF4A antisera recognise both eIF4A1 and eIF4A2 proteins (Fig. S1), we repeated the affinity purification and probed with anti-eIF4A2 affinity-purified antibodies in order to determine whether this isoform of eIF4A is sumoylated. In this case, a species of ∼150 kDa is observed with both His–SUMO1 and His–SUMO2 (Fig. 1Biv), likely corresponding to the species of ∼150 kDa observed with anti-eIF4A antisera. These results indicate that, like eIF4E and eIF4G, eIF4A2 is sumoylated *in vivo*, and that it is modified by both SUMO1 and SUMO2. The levels of sumoylation of these initiation factors are typically in the region of 2–5% total eIF4A2 (similar to sumoylation levels reported for other proteins, e.g. Johnson and Blobel, 1999).

### Sumoylation of eIF4A2 is increased in response to arsenite and ionising radiation

**Fig. 2.**
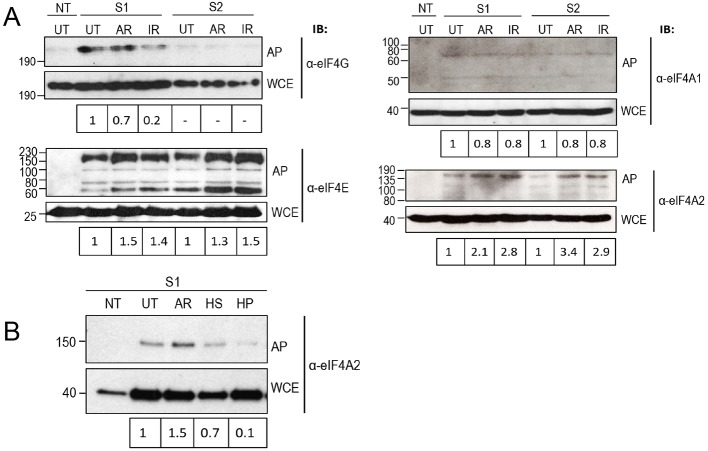
**Exposure to arsenite and ionising radiation affects sumoylation of eIF4G, eIF4E, eIF4A1 and eIF4A2.** Whole-cell extracts (WCE) and affinity purification under denaturing conditions as in Fig. 1 (AP) of His-tagged SUMO1 (S1) or SUMO2 (S2) from stably transfected HeLa cell lines or non-transfected cells (NT), untreated (UT) or subjected to 1 mM arsenite (AR) for 30 min or 3 Gy ionising radiation (IR) with 30 min recovery, analysed by SDS-PAGE (7.5%) and western blotted. (A) Western blots probed with antisera against eIF4G and eIF4E under conditions used in Fig. 1B, as indicated, and with affinity-purified antibodies against eIF4A1 and eIF4A2 as indicated. (B) Whole-cell extracts (WCE) and affinity purification (AP) as in Fig. 1A from cells that were exposed to 1 mM arsenite, heat shocked at 42°C for 30 min (HS) or treated with 1 μM hippuristanol (HP) for 60 min. Samples were analysed by western blotting as in A.

We have previously demonstrated that *Schizosaccharomyces* *pombe* eIF4G is sumoylated in response to 1 M KCl (a condition that induces stress granules in yeast) (Jongjitwimol et al., 2014). We therefore investigated the effect of stress on sumoylation of eIF4G, eIF4E or eIF4A2 in mammalian cells. We began by investigating the effect of arsenite, which induces the formation of stress granules, and ionising radiation, which causes genotoxic stress. Analysis of global levels of sumoylation in response to arsenite (1 mM) and ionising radiation (3 Gy) (Fig. S2A), indicated that exposure to arsenite had a minimal effect on the levels of sumoylation by either SUMO1 or SUMO2. In contrast, exposure to ionising radiation reduced the level of global sumoylation by ∼50%. To analyse sumoylation of the individual eIFs, His–SUMO1 and His–SUMO2 were affinity-purified from untreated cells and cells exposed to arsenite or ionising radiation. Fig. 2A shows that sumoylation of eIF4G by SUMO1 was somewhat reduced following exposure of cells to arsenite. In contrast, sumoylation by SUMO1 was reduced in response to ionising radiation. In comparison to sumoylation by SUMO1, only low levels of SUMO2-containing species were observed. In the case of eIF4E, sumoylation by both SUMO1 and SUMO2 increased in response to both arsenite and ionising radiation (Fig. 2A). All four species seen represent sumoylated forms of eIF4E; because the significance of the four different forms is unknown, we have compared the levels of the total amount of sumoylated species here.

We next investigated whether sumoylation of the eIF4A1 and eIF4A2 isoforms was affected by stress. Analysis of the sumoylated species of eIF4A1 indicated that only very low levels of sumoylated eIF4A1 were observed under normal conditions, and that the levels of these species were not altered in response to arsenite or ionising radiation (Fig. 2A). In contrast, levels of sumoylated eIF4A2 increase in response to both stresses (Fig. 2A). This increased sumoylation in response to arsenite and ionising radiation resembles that of eIF4E suggesting that sumoylation of these two factors might be coordinately regulated.

**Fig. 3.**
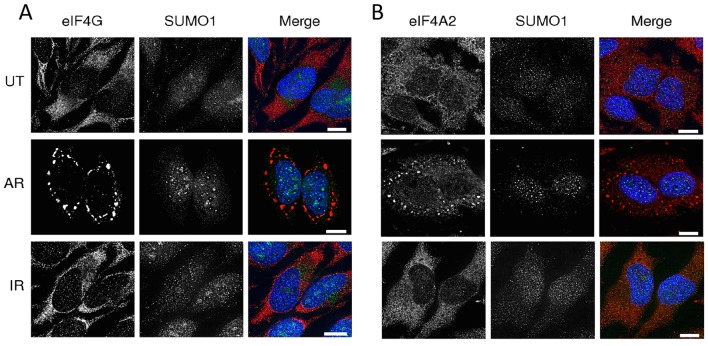
**Exposure to arsenite affects the localisation of eIF4A2 and eIF4G.** HeLa cells were cultured and treated as in Fig. 2, and were either treated with 1 mM arsenite (AR) for 30 min or exposed to 3 Gy ionising radiation (IR) and allowed to recover for 30 min. Cells were then fixed and stained for SUMO1 and either eIF4A2 or eIF4G. NT, non-transfected cells. (A) Immunofluorescence staining was performed using eIF4G and SUMO1. (B) eIF4A2 and SUMO1. Merged images show DAPI (blue), eIF4A2 or eIF4G (red), and SUMO1 (green). Scale bars: 10 μm. We have observed at least 100 cells in each of three replicates and clearly see that all cells exposed to arsenite contain stress granules.

**Fig. 4.**
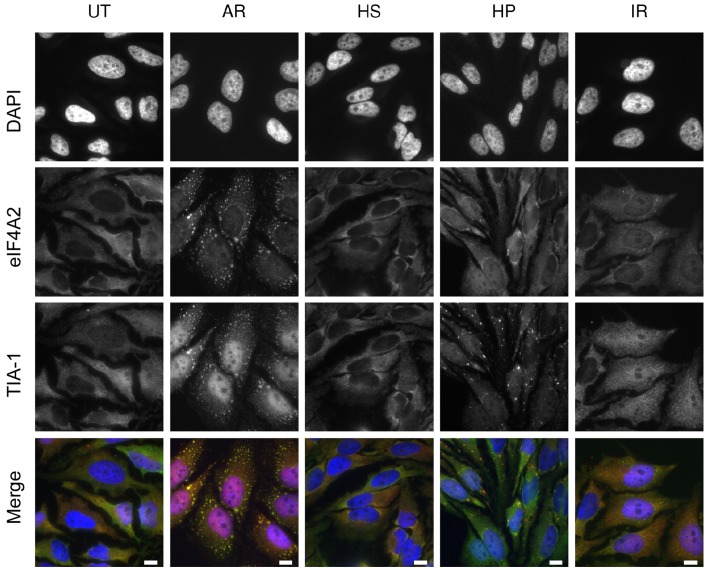
**eIF4A2 localises to arsenite-induced stress granules but not to hippuristanol-induced stress granules.** HeLa cells were cultured and either left untreated or subjected to the following treatments, 1 mM sodium arsenite (AR) for 30 min, heat-shock (HS) at 42°C for 30 min, 1 μM hippuristanol (HP) for 60 min or 3 Gy ionising radiation (IR) recovery for 30 min. The cells were fixed and immunostained for both eIF4A2 (as in Fig. 3) and TIA-1, followed by DAPI. Merged images show TIA-1 (red), eIF4A2 (green) and DAPI (blue). Scale bars: 10 μm.

Sumoylation of proteins is known to affect protein localisation (e.g. Muller et al., 1998; Finkbeiner et al., 2011). Given that exposure of cells to arsenite results in the relocalisation of certain translation initiation factors to stress granules (Kedersha et al., 1999), we wished to determine whether sumoylation of eIF4A2 was correlated with stress granule formation. We therefore compared the effects of arsenite and ionising radiation on the localisation of SUMO, eIF4G and eIF4A2. In untreated cells, the majority of the SUMO was present in small nuclear speckles in the nucleus, with low levels in the cytoplasm (Fig. 3). In cells treated with arsenite, SUMO was relocalised to intra-nuclear foci, known as promyelocytic leukaemia (PML) bodies, as has been demonstrated previously (Muller et al., 1998), with low levels remaining in the cytoplasm, whereas exposure to ionising radiation resulted in small nuclear foci. In untreated cells, eIF4G and eIF4A2 were predominantly in the cytoplasm, but with a substantial proportion in the nucleus (Fig. 3). Arsenite treatment resulted in the relocalisation of both eIF4G and eIF4A2 into cytoplasmic granules. Staining of arsenite-treated cells with antibodies against TIA-1, a known stress granule marker (Kedersha et al., 1999), identified these as stress granules (Fig. 4). In contrast to the effect of arsenite on eIF4A2 and eIF4G localisation, exposure to ionising radiation had little effect on the localisation of eIF4G and eIF4A2 (Fig. 3).

### Sumoylation of eIF4A2 is reduced following heat shock and exposure to hippuristanol

We next extended our studies to analyse sumoylation of eIF4A2 in response to two other stresses: heat shock and hippuristanol. Hippuristanol is a small-molecule inhibitor of eIF4A1 and eIF4A2 that has previously been demonstrated to induce the formation of eIF4A1-containing stress granules in an eIF2α-phosphorylation-independent manner (Mazroui et al., 2006). Given that modification of eIF4E and eIF4A2 by SUMO1 and SUMO2 appeared to be similar, we focused here on sumoylation by SUMO1. Analysis of global sumoylation levels of SUMO1-containing species indicated that there was an increase in response to heat shock, but a slight reduction in response to hippuristanol (Fig. S2B). As described previously, His–SUMO1 was purified from cells under denaturing conditions and western blots were probed with anti-eIF4A2 antibodies. Fig. 2B indicates that, in contrast to the increased levels of sumoylation we observed with arsenite, sumoylation by SUMO1 of eIF4A2 was reduced following exposure to heat shock or hippuristanol.

As shown in Fig. 3, increased sumoylation of eIF4A2 correlated with its relocalisation to stress granules. We therefore investigated whether reduced sumoylation of eIF4A2, for example, as in the case with hippuristanol, had any effect on this repositioning. Furthermore, we wished to confirm that the aggregates we observed were indeed stress granules. We therefore repeated the immunofluorescence studies to compare the effects of arsenite, heat shock, hippuristanol and ionising radiation using anti-eIF4A2 antibodies, along with anti-TIA-1 antibodies to identify stress granules. Fig. 4 indicates that in response to arsenite, eIF4A2 colocalised with TIA-1, confirming the relocalisation of eIF4A2 into stress granules, an effect not observed with heat shock under our assay conditions. As expected, exposure of cells to hippuristanol resulted in the formation of stress granules; however, these did not contain eIF4A2. This further supports the notion that sumoylation of eIF4A2 correlates with its ability to form stress granules.

### Identification of the sumoylation sites on eIF4A1 and eIF4A2

**Fig. 5.**
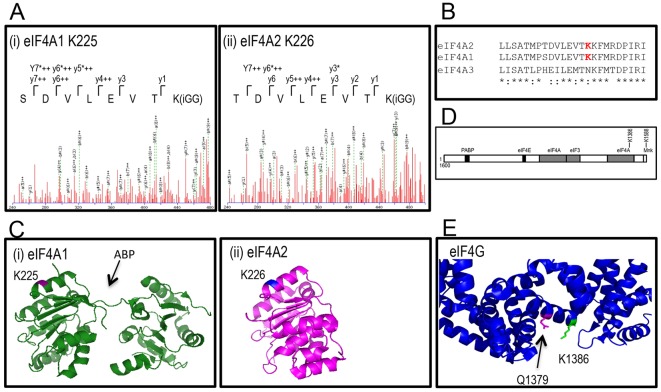
**eIF4A1 and eIF4A2 are sumoylated on K225 and K226, respectively.** (A) Mass spectra of sumoylation products. High-molecular-mass species from an *in vitro* sumoylation assay were excised from SDS-PAGE gels and analysed by LC-MS/MS. (B) Sequence alignment of human eIF4A1, eIF4A2 and eIF4A3. Sumoylation sites are indicated in red. (C) Position of sumoylation sites on eIF4A1 (i) and eIF4A2 (ii). Pymol-derived figure indicating sumoylation sites on human eIF4A1 (PDB ID 2ZU6) and eIF4A2 (PDB ID 3BOR). ABP, ATP-binding pocket. (D) Schematic indicating organisation of interacting motifs in eIF4G. (E) Positions of sumoylation site (K1386, green) and Q1379 (magenta) in the C-terminal fragment of eIF4G (PDB IB 1UG3).

In order to identify the lysine residues used as SUMO-acceptor sites on eIF4A1 and eIF4A2, we used an *in vitro* sumoylation assay previously established in our laboratory (Ho et al., 2001). Recombinant eIF4A1 and eIF4A2 were incubated with purified sumoylation components, with a modified form of SUMO containing a trypsin cleavage site adjacent to the diglycine motif (Jongjitwimol et al., 2014), and products were analysed by liquid chromatography tandem mass spectrometry (LC-MS/MS). In both cases, a single lysine residue was identified: K225 and K226, respectively (Fig. 5A). The eIF4A1 and eIF4A2 proteins are highly conserved, as is the region containing the sumoylation sites (Fig. 5B). This is not the case with eIF4A3, which does not have a lysine residue at the equivalent position. Modelling of the sumoylation sites onto the known crystal structures indicated that the sites are in similar positions in the two proteins (Fig. 5C) on a highly accessible surface of the protein in an α-helical domain. These sites face the ATP-binding pocket (Oberer et al., 2005), suggesting that sumoylation of eIF4A might affect its interaction with ATP.

We have previously demonstrated that the C-terminus of mammalian eIF4G is also sumoylated. Two sites were identified: K1386 and K1588. These residues map to the eIF4A- and mitogen-activated protein kinase (MAPK) interacting protein kinase 1 (Mnk1)-interacting regions of eIF4G (Fig. 5D). Molecular modelling indicated that, interestingly, K1386 is close to Q1379 (Fig. 5E), which when mutated to lysine results in increased binding of eIF4G to eIF4A (Bellsolell et al., 2006).

### eIF4A2 is sumoylated on K226 *in vivo*

**Fig. 6.**
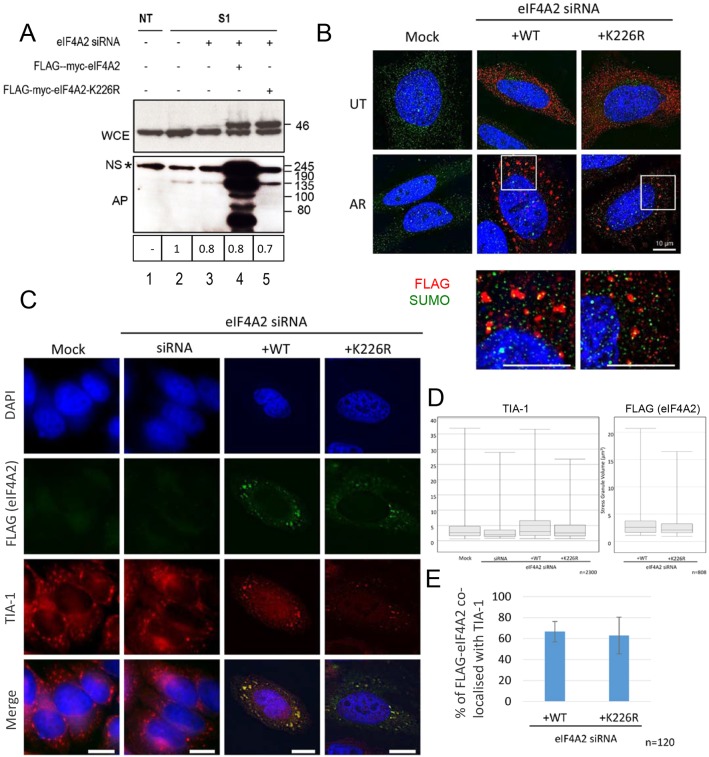
**Mutation of K226 results in loss of sumoylation of eIF4A2 *in vivo* and a reduction in stress granule size.** (A) His–SUMO1 stably transfected cells were reverse transfected with eIF4A2 siRNA (lanes 3–5). After 48 h, cells were mock treated (lane 2) or transfected with FLAG–myc–eIF4A2 wt or FLAG–myc–eIF4A2-K226R mutant as indicated. His-SUMO1 was purified from non-transfected cells (NT, lane 1) or His–SUMO1 stably transfected cells (S1, lanes 2–5) using Ni^2+^ agarose under denaturing conditions as in Figs 1 and 2. Proteins were analysed by SDS-PAGE and western blotted with anti-eIF4A2 antisera. (B) HeLa cells were depleted of endogenous eIF4A2 and transfected with either FLAG–myc–eIF4A2 wt or FLAG–myc–eIF4A2-K226 and either left untreated (UT) or treated with 1 mM arsenite (AR) for 30 min. Cells were immunostained with an anti-FLAG antibody and anti-SUMO. High-resolution images (lower panels) were taken over a *z*-plane of 4 μm at 0.05 μm slices. Scale bars: 10 μm. (C) HeLa cells were prepared as in B and immunostained for FLAG (eIF4A2) and TIA-1. Scale bars: 10 μm. (D) High-resolution *z*-stack images were taken and deconvolved using Huygens deconvolution software. Images were three-dimensionally rendered using the IMARIS software suite. 3D images were used to calculate the volumes of stress granules for both TIA-1 and FLAG (eIF4A2). Box-and-whisker plots were used to show the distribution of stress granule volumes. The box represents the 25–75th percentiles, and the median is indicated. The whiskers show the 10–90th percentiles. *n*=2300 for TIA-1 and 800 for FLAG. (E) Cells processed as in C were analysed for colocalisation of FLAG and TIA-1 signals. The chart shows the percentage of FLAG (eIF4A2) signal that overlaps with TIA-1 in cells transfected with either FLAG-eIF4A2 WT or the K226R mutant (mean±s.d., *n*=120).

Given that eIF4A2 is more highly sumoylated *in vivo* than is eIF4A1 (Fig. 2A), we concentrated our studies on the eIF4A2 isoform, focusing on SUMO1-containing species, as this would cover singly sumoylated species as well as those containing SUMO chains terminating with SUMO1. Having shown that eIF4A2 is sumoylated *in vitro* on K226, we wished to confirm that this residue is used for sumoylation *in vivo*. To do this, expression of endogenous eIF4A2 was knocked down using small interfering RNA (siRNA), and cells were then transfected with wild-type (wt) or mutant eIF4A2 (Fig. 6A). Despite the fact that the knockdown was only partial (eIF4A2 is a very abundant protein making it difficult to achieve 100% knockdown), introduction of siRNA-resistant wild-type eIF4A2 into cells clearly results in new high-molecular-mass species being observed following affinity-purification of His-SUMO1 (lane 4). These species are of similar molecular mass to those observed in Figs 1B and 2B, and are not observed in the untransfected controls (lanes 1–3). In contrast, although it is clear that the eIF4A2-K226 protein was expressed at similar levels to the wt protein (lanes 4 and 5, lower panel), such species were not observed when the eIF4A2-K226R mutant is introduced (lane 5). This indicates that K226 is the main sumoylation site in eIF4A2 that is used *in vivo*.

### Inability to sumoylate eIF4A2 results in a reduction in stress granule volume

To determine whether the inability to sumoylate eIF4A2 affects either its recruitment to stress granules or the formation of the granules, we used immunofluorescence with anti-FLAG antibodies to compare the localisation of FLAG-tagged wt and FLAG–eIF4A2-K226R protein expressed in cells as described above (Fig. 6B). In unstressed cells, the localisation of the wt and mutant proteins were very similar and resembled what we observed for endogenous eIF4A2 (Fig. 3B). However, there was a distinct difference in the localisation of the wt and mutant proteins in response to arsenite. As observed with the endogenous protein, FLAG-tagged eIF4A2 was observed in peri-nuclear stress granules, indicating that the tag did not affect localisation. However, recruitment of FLAG–eIF4A2-K226R to stress granules was substantially less than that of the wt eIF4A2. To confirm the identity of the granules, we repeated these experiments using anti-TIA-1 antibodies in combination with anti-FLAG antibodies. In arsenite-treated cells where eIF4A2 had been knocked down, there was a substantial reduction in TIA-1-containing stress granules, particularly in the number of large granules, compared to the situation in mock-treated cells (Fig. 6C). Transfection with FLAG–eIF4A2 wt restored the ability of cells to produce TIA-1-containing granules in response to arsenite treatment. In cells transfected with FLAG–eIF4A2-K226R, stress granules were also observed, although there were generally fewer granules per cell. In particular there was a substantial reduction in the number of large TIA-1-containing stress granules (Fig. 6D), similar to the situation observed in eIF4A2-knockdown cells. This reduction in large TIA-1-containing stress granules correlated with the reduction in the number of large eIF4A2-containing granules. Furthermore, in cells transfected with either wt or mutant eIF4A2, there was a high degree of colocalisation of eIF4A2 (both wt and mutant) with TIA-1 (Fig. 6E).

Despite the inability of eIF4A2 to be sumoylated, low levels of SUMO were still detected in stress granules in cells expressing FLAG–eIF4A2-K226R (Fig. 6B, bottom panels). This is likely to be due to the fact that other components in stress granules are also sumoylated, for example, eIF4G (Jongjitwimol et al., 2014; Bish et al., 2015). These results indicate that it is the inability of eIF4A2 to be sumoylated, rather than the absence of SUMO that is causing the defect in stress granule formation.

## DISCUSSION

Stress granules have been characterised as cytoplasmic aggregates that contain a number of proteins, including translation initiation factors, ribosomal subunits, PABP and TIA proteins. Among the translation initiation factors that are recruited to stress granules are eIF4G and eIF4A, and more specifically eIF4A1 (Mazroui et al., 2006). Here, we demonstrate that another isoform of eIF4A, eIF4A2, which is functionally distinct from eIF4A1 (Lu et al., 2014), can also be recruited to stress granules.

It has been well documented that post-translational modifications are required for stress granule assembly (Buchan and Parker, 2009). For example, a key event required for this process is phosphorylation of eIF2α, and mutation of HDAC6, a histone deacetylase that impairs stress granule formation (Kwon et al., 2007), implicating acetylation as being involved. Additionally, stress granules have been demonstrated to contain ubiquitin-modified proteins (Kwon et al., 2007). Our studies here suggest that sumoylation also has a role in protein recruitment to stress granules. Specifically, we demonstrate that although arsenite and hippuristanol both induce the formation of stress granules, only in cells exposed to arsenite, which increases the level of sumoylation of eIF4A2, is eIF4A2 recruited to stress granules. In contrast, in hippuristanol-treated cells where sumoylation of eIF4A2 is decreased, there is decreased recruitment of eIF4A2 to stress granules. Unlike eIF4A2, eIF4A1 is recruited to stress granules in response to hippuristanol (Mokas et al., 2009), further supporting the view that the two proteins are functionally distinct (Lu et al., 2014).

Using a combination of *in vitro* and *in vivo* studies, we have demonstrated that eIF4A2 is sumoylated on K226. Mutation of this site results in the impaired formation of TIA-1-containing stress granules, as demonstrated by a decrease in overall stress granule volume after treatment of cells with arsenite, thus indicating a role for sumoylation in the formation of these granules. The sumoylation site on eIF4A2 is on the same face as the ATP-binding site suggesting that sumoylation might affect the interaction of eIF4A2 with ATP or its ATPase activity. It is known that ATP binding by eIF4A causes a switch in the protein to a closed conformation that allows ATP hydrolysis (Marintchev et al., 2009). This subsequently facilitates the formation of an open conformation, resulting in reduced affinity of eIF4A for mRNA. Thus, inability to sumoylate eIF4A2 might affect its interaction with mRNA, which in turn could disrupt stress granule formation. Interestingly, hippuristanol, which inhibits the recruitment of eIF4A2 to stress granules, is known to interact with the eIF4A helicases at the ATP-binding site, and inhibit the ATPase, RNA-binding and helicase activities of the protein (Lindqvist et al., 2008). This supports the notion that sumoylation of eIF4A2 might be affecting its interaction with ATP and/or RNA binding.

Molecular modelling indicates that one of the sumoylation sites in eIF4G that we have identified (K1386) maps to a region involved in interacting with eIF4A (Fig. 5D). This further suggests that sumoylation might regulate the interaction between eIF4G and eIF4A. Indeed, mutation of a nearby residue (Q1379 to lysine) results in increased binding of eIF4G to eIF4A (Bellsolell et al., 2006). Given that this lysine residue could potentially be targeted by sumoylation, it would be interesting to determine whether the same result is achieved by mutation to arginine (still a charge reversal mutation, but to a residue which does not act as a SUMO acceptor).

As well as interacting with eIF4G, eIF4A2 interacts with cNOT7, where it is likely involved in miRNA-mediated translational repression. In collaboration with Lu and Bushell (University of Leicester, Leicester, UK), we have observed using overexpression constructs that mutation of the eIF4A2 sumoylation site (K226) has no effect on this process (Lu and Bushell, personal communication). This would be consistent with miRNA-mediated translational repression being a process used under normal growth conditions, rather than a stress response, a condition where we see increased sumoylation of eIF4A2.

Identification of the effect(s) of sumoylation on individual proteins has, in many cases proved problematic. For example, there are a number of well-studied cases where several members of multiprotein complexes are all sumoylated (Jentsch and Psakhye, 2013; Nie et al., 2015). In such cases, inability to sumoylate a single member of the complex has little affect because sumoylation of other components of the complex is sufficient for function. Thus, although we have shown that sumoylation of eIF4A2 is required for efficient formation of stress granules, it is possible, because this protein is present in eIF4F complexes that contain eIF4G and eIF4E, both of which are also sumoylated, that there might be further roles for sumoylation of eIF4A2 still to be uncovered. However, now that the sumoylation sites on the members of the eIF4F complex have been identified, it should be possible to obtain a fuller understanding of the role of sumoylation in regulating translation initiation.

## MATERIALS AND METHODS

### Protein expression and purification

Recombinant His–eIF4A1 and GST–eIF4A2 were prepared from *E. coli* BL21 cells using Ni^2+^ agarose (Novagen) and glutathione–Sepharose, respectively, according to the manufacturer's instructions. Mammalian whole-cell extracts (WCEs) were prepared from 5×10^5^–10^6^ mammalian cells. Cells were washed once with PBS and then resuspended in 500 µl of ice-cold 0.24 M NaOH and 1% (v/v) β-mercaptoethanol. The mixture was incubated on ice for 15 min. 75 µl of 50% trichloroacetic acid (TCA) was then added to the lysate that was further incubated on ice for 10 min. Denatured proteins were precipitated by centrifugation at 13,000 ***g*** for 20 min at 4°C, and then resuspended in 30 µl of 1× SDS sample buffer. His-tagged SUMO was affinity-purified from HeLa cells under denaturing conditions using Ni^2+^ agarose as described previously (Jongjitwimol et al., 2014). Western blotting was carried out as described previously (Harlow and Lane, 1988).

### Identification of sumoylation sites

The *in vitro* sumoylation assay was carried out as described elsewhere (Ho et al., 2001). A modified form of SUMO containing a trypsin cleavable site adjacent to the C-terminal diglycine motif, used for mass spectrometric analysis, was as described previously (Jongjitwimol et al., 2014). Samples were prepared for mass spectrometry using a modification of the method of Shevchenko et al. (2006), as described previously (Jongjitwimol et al., 2014).

### Molecular modelling

The positions of sumoylation sites on eIF4A and eIF4G crystal structures were located using Pymol.

### Tissue culture, cell lines and reagents

HeLa cells (supplied by ATCC, validated on 14 May 2015 and demonstrated to be mycoplasma-free on 27 May 2015) were cultured in DMEM supplemented with 10% (v/v) fetal calf serum (FCS), penicillin, streptomycin and L-glutamine. HeLa cells stably transfected with His-tagged SUMO1 and SUMO2 were gifts from Ron Hay (University of Dundee, Dundee, UK) (Girdwood et al., 2003; Vertegaal et al., 2004). For analysis of stress responses, cells were treated with 1 mM sodium arsenite for 30 min, 3 Gy ionising radiation from a ^137^Cs source and left to recover for 30 min, heat shock at 42°C for 30 min or 1 μM hippuristanol for 60 min.

### siRNA depletion of eIF4A2 and siRNA-resistant expression of eIF4A2

The K226R mutation was introduced into siRNA-resistant eIF4A2 by site-directed mutagenesis using the QuickChange method (Stratagene). siRNA (Silencer Select s4572) was from Life Technologies as described previously (Meijer et al., 2013), and the siRNA-resistant FLAG-myc-eIF4A2 construct was a gift from Martin Bushell (MRC Toxicology Unit, University of Leicester, UK) (Meijer et al., 2013).

### Antibodies

Antibodies used were as follows with dilutions in parentheses. Rabbit anti-eIF4G (1:10,000), anti-eIF4E (1:3000) and anti-eIF4A (1:2000) were as described previously (Coldwell et al., 2012; Morley and Pain, 1995). Mouse monoclonal anti-eIF4G (sc-373892; 1:3000 for western blotting, 1:50 for immunofluorescence), rabbit polyclonal anti-SUMO1 antibody (sc-9060; 1:2500 for western blotting, 1:50 for immunofluorescence), mouse monoclonal anti-SUMO1 (sc-5308; 1:2500 for western blotting, 1:50 for immunofluorescence), rabbit polyclonal antibody against both SUMO2 and SUMO3 (sc-32873; 1:2500) were from Santa Cruz Biotechnology. Mouse monoclonal anti-FLAG (F1804; 1:1000 for western blotting, 1:400 for immunofluorescence) was from Sigma-Aldrich. Goat polyclonal anti-eIF4A1 (sc-14211; 1:1000) and mouse anti-eIF4A2 (sc-137147; 1:200 for immunofluorescence) were from Santa Cruz Biotechnology. Rabbit anti-eIF4A2 (Ab194471; 1:3000) and rabbit anti-TIA-1 (Ab40693; 1:400) were from Abcam. Secondary antibodies used were: horseradish peroxidase (HRP)-conjugated goat anti-rabbit-IgG and rabbit anti-mouse-IgG (Dako), both used at 1:2500 for western blotting, FITC-conjugated anti-mouse-IgG and TritC-conjugated anti-rabbit-IgG (Sigma-Aldrich), Cy5-conjugated anti-rabbit-IgG (Life Technologies) and TritC-conjugated anti-goat-IgG (Santa Cruz Biotechnology) all used at 1:200.

### Immunofluorescence

Cells cultured on coverslips were fixed using 4% (w/v) PFA in PBS for 10 min and permeabilised using 0.1% (v/v) Triton X-100 in PBS for 30 s. Cells were washed three times with PBS before incubation for 1 h at room temperature with the primary antibodies diluted to the indicated concentration in 4% (w/v) BSA in PBS. Cells were washed a further three times with PBS before incubation for 30 min with the secondary fluorophore-coupled antibodies. Secondary antibodies were diluted as above in 4% (w/v) BSA in PBS. Cells were washed a final three times before mounting on glass slides with ProLong Gold with DAPI mounting medium (Life Technologies). Cells were visualised using an Olympus IX70 microscope. Stress granules were analysed by taking deconvolved high-resolution *z*-stack images over 4 µm at 0.05 µm intervals. Imaris software suite was used to render three-dimensional images. Surface mapping was used to create and calculate stress granule volumes.
